# Supplementary material for: Kinetic Modeling and Meta-Analysis of the Bacillus subtilis SigB Regulon during Spore Germination and Outgrowth
Source: Microorganisms. 2021 Jan 5;9(1):112. doi: 10.3390/microorganisms9010112 (PMC7824861; doi:10.3390/microorganisms9010112)
Supplement: Supplementary file 1 [file microorganisms-09-00112-s001.zip › microorganisms-1052470 -s/Supplementary file 2 Table primers.docx]

**Supplementary file 3. List of primers**

Primer names contain the serial number in the catalogue and the name of the gene. F – forward primer. R – reverse primer. Short/long versions are indicated. The hcrA DNA fragments were used to generate molecular size RNA marker of 201, 253, 300, 357, and 407 nt, respectively.

| **Primer name** | **Sequence (5' → 3')** |
| --- | --- |
| 2088/hrcA_F | GTCGGACCTGTTAAGCATTAC |
| 2089/hrcA_R | CGTTGATTATAACCTGAAGG |
| 2110/PhrcA_R2 | CAATTCCTCCAAGTCAGCCATC |
| 2111/PhrcA_R3 | CTTTTTCTGACGGAACACGTCC |
| 2112/PhrcA_R4 | CGTCAATTTGACGGGTGACAGC |
| 2113/PhrcA_R5 | CTCCAGCTCGAAAATTTTCTC |
| 2114/PhrcA_R6 | GTGTAATTCGTCAGATCGGAC |
| 1069/trxA_F | ccgGAATTCattccggagtcattcttacgg |
| 3079/trxA_R_short | cctgatcacagccggtttac |
| 3080/trxA_R_long | catctcccattcgttcacgc |
| 3036_pgcA_F | ATGAGGCCGCGAAACGCAAC |
| 3037_pgcA_R_long | GCCAGCTGCCTCCTGATAAC |
| 3098/pgcA_R_short | GCCTCTGTCACTTGATAACC |
| 3038_yjlB_F | GCCAGCTGCCTCCTGATAAC |
| 3039_yjlB_R_short | AATCAGGGTGATTCGGAATC |
| 3081/yjlB_R_long | GTCCGGTGTCCTTAAGCGCG |
| 3040_nhaX_F | GCGGTCCGACCTAAAAAACC |
| 3041_nhaX_R_short | GTTTTAGCAAGATCAATGGC |
| 3082/nhaX_R_long | TGGGCGACGGTAATAGCGGC |
| 3046_yflT_F | TAGACATACCTCCTGCTCGT |
| 3047_yflT_R_short | GGTCAAGATCCCCTACGAAC |
| 3084/yflT_R_long | GTTCTGTCATCGTCGTGAGC |
| 3050_ctc_F | GCTTGTTGTGACAAACAGCA |
| 3051_ctc_R_short | TACGGATATTCCGAAGAGAC |
| 3086/ctc_R_long | CCGTATCCTTCCCATATATG |
| 3052_phoH_F | CAATCCCGATCTGGGGGATG |
| 3053_phoH_R_short | TCTCCCTGACAATAGGTGTG |
| 3087/phoH_R_long | TCGCAAGTAAATGTTCTGTC |
| 3044_yqhY_F | GCTGATTTCGCTGAATTATG |
| 3045_yqhY_R_short | CGTGGCGAAAATCTTCTATG |
| 3083/yqhY_R_long | GTATTTCCGTAGTTATCAGC |
| 3062_yvyD_F | CCTCTCATGAGTTCTGTGAG |
| 3063_yvyD_R_short | TCCTTTAACGCGGGTGTCAC |
| 3088/yvyD_R_long | AGCGCTCCAGCTTGCCGATC |
| 3064_glyA_F | CAGCCTCGGTTCAGCTCATG |
| 3065_glyA_R_short | ATCTTAGTCTGTTGGCGTTC |
| 3089/glyA_R_long | CCGCTTCACTTACAAAGTTC |
